# Ready Rubber

**Published:** 1871-02

**Authors:** 


*Ready Rubber.*—In reply to numerous inquiries made concerning this preparation, and also of its modification, as well as the terms upon which they are offered to the dental profession, we publish the following paper prepared by Dr. Sam'l S. White, who has spent much time and money in the employment of counsel and experts, in trying to find out if there was any relief from the Goodyear and Cumming's patents, or any merit in the "Ready Rubber" for dental purposes:

"I understand the "Ready Rubber," which heads Mr. Isaac H. Herbert's circular, to be a patented compound material or substance, consisting of caoutchouc, turpentine, sulphuric acid, and a mode of treating these ingredients, as described in John B. Newbrough's Patent of March 1, 1870, numbered 100,435, designated as "a new process for hardening Rubber with a compound known as Acid Resin."

The circular states that this compound "is furnished already vulcanized hard, and can be remelted and moulded in any shape or form, and as soon as cold is perfectly hard."

The license offered to dentists is to "use said rubber manufactured under said patent."

Of what constitutes the "modification of the above," referred to in the same circular, I have no knowledge beyond a verbal statement made to me by Mr. Herbert, that it is a mixture of Whalebone and Ready Rubber; but as it requires vulcanization by the dentist, "the same as Whalebone Rubber," it is a different preparation from that for which they offer a license.

I have had submitted to me, and have examined and tested, in the presence of their representative, what were called samples of "Ready Rubber."

The result of my attention to the subject is, that I have not yet seen a specimen of the product fit for dental uses. Neither were the specimens of mixed Whalebone and Ready Rubbers, called a "modification," furnished by Mr. Herbert, fit for dentists' use. They are confessedly unfit; but they promise to make them good.

In regard to the terms proposed: they ask from dentists, apart from, or in addition to, a higher price for the material, a license fee or purchase of office right, during the term of their patent. By this I understand that the sum named for "office right" is the price of their guarantee and protection.

The license grants a right "to use the said rubber manufactured under said patent," but it does not grant the right to use said patent in making Ready Rubber should they fail to supply it.

This has to my mind the same objection that obtained against the Boston Dental Vulcanite Co.'s use of the license system to a degreee certainly oppressive, and by many considered as taking an illiberal and unjust advantage of their necessities.

Regarding the promise to "defend" their licenses, and their responsibilities on that promise; I do not know who are "the owners of said patent," and neither circular nor printed license binds the owner or gives any information on that subject. I note particularly, however, that while their license binds the dentist not to defend any suit brought against him for the use of their rubbers, they leave him without recourse or guarantee if they default or are defeated, and personally liable for costs and damages.

That the owners of the Goodyear and Cummings patent will attack just when it suits them to do so; that they will not consult the interest or convenience of the "Ready Rubber" party, or of those working under their license, there can be no doubt. In addition to this, I think—as their standing in court now is—that the owners of these patents can get preliminary injunctions at once against any dentist using any "Vulcanized Rubber," or constructing a plate of material requiring similar processes. Whether the "Ready Rubber" party will promptly defend; whether they will succeed in that defence; or whether their "guarantee" and protection will prove efficient, time alone will determine, as it will also determine the qualities, of "Ready Rubber," and the "Modification" of it, and their fitness for dental uses.

Therefore while it would afford me great pleasure to announce an article of equal value to hard rubber, free from the illiberal exactions and offensive modes of settlement that have caused the Boston Dental Vulcanite Company, represented by Josiah Bacon, to be a stench in the nostrils of the dental profession, and hating even in this indirect manner to aid the continuance of their exactions, I am extremely sorry that a long and careful investigation leaves me without confidence that any relief can be found in this direction."

